# With Modern Eyes

**Published:** 1892-04-30

**Authors:** 


### THE DOCTOR'S ARMCHAIR.

WITH MODERN EYES.

Most people who are now living have been born within the nineteenth century; but he would be a very inexperienced man who should say that we all belong to the nineteenth century. The eye sees easily what it is trained to see; it may often miss seeing altogether something which to other eyes is as manifest as the moon or the stars on a frosty night. Some people pride themselves upon the possession of an eye that sees best, or sees only what is ancient; others cannot look upon the ancient with patience; their eyes are for what is modern, and for nothing else. The best eye, after all, is that which sees all kind of things, both ancient and modern, and sees them as they are, in their true measure and value. To deny this is to put a premium upon occasional blindness. Now blindness, which is even occasional, may lead to all manner of fatalities.

A painter passing through an English village sees a cottage with thick, moss-covered walls, small windows with tiny diamond panes, a thatched roof, and a chimney from which the light smoke curls upwards; and he is filled with enthusiasm for its picturesque beauty. He looks at it with eyes that are as old as art. A modern physiologist passes by, and he, too, sees the cottage. He is, perhaps, conscious, as the painter was, of its picturesqueness, but he is conscious also of much more. The building to him is a place for men and women to live in, for children to be born and brought up in; for the aged to die in. He looks at it, therefore, as the soldier looks at his rifle—from the point of view of the use to which it is to be put. It is evident to him that the walls are not only thick and moss-grown, but damp, soaking with the moisture of ages; the windows are so small that they let in neither air nor light, the ceilings are low, and the roof is rotten.

Here is a description of some cottages as they now actually exist at Scotter, on the banks of the Trent, in North Lincolnshire. These cottages, it will be apparent, have none of the picturesqueness which delights the artist, but more than their due share of the ruinous unhealthiness which excites the indignation of the patriotic physiologist. The writer of the description is Dr. Franklin Eminson, Medical Officer of Health for the Scotter district of the Gainsborough Union. Says Dr. Eminson: "The houses in Scotter village number about 180; they are mostly built of common brick, often of such porous nature that rain readily permeates the nine-inch walls, making the bed-rooms damp and the wall-papers dank and mildewed. Most of them have been built without under-draining, and the foundations laid without slate or other damp proof course, and as the foundations in the lower parts of the village are often beneath the level of the subsoil water, the walls are excessively damp, moisture rising in some instances nearly as high as the bed-rooms. Nearly forty per cent. are without spouting, and the rain-water, dropping close to the foundations, hollows out the ground, and lies in pools, making the walls and ground floors damper than they would otherwise be. Where spouting does exist, it is frequently rotten and grass-grown, the water running down the walls and into the cracks and crevices."

The present writer, happily for himself, has, by much familiarity with facts of this kind, got beyond the stage of what Carlyle called "Fire-eyed indignation." But a just indignation is, or ought to be, succeeded by practical determination: a determination, first to take in the full significance of such facts; and secondly to do whatever may be possible to get such disgraceful things put an end to for ever. Let us hear, from Dr. Eminson's own mouth, of some of the consequences that have come upon the people of Scotter as the result of their ill-built houses, badly-drained streets, and the want of the "modern eyes" which see these things in their true light. We will take at random a few extracts from a pamphlet issued by Dr. Eminson, which deals with the "Disease History of Scotter" generally, and with a recent "Epidemic of Pneumonia" in particular. For example: "Scurvy is believed to have been common in Scotter and its neighbourhood in old times." . . . "Ague was formerly prevalent in the neighbourhood." . . . "Small-pox was once an exceedingly common and fatal disease in Scotter." . . . "Up to ten or fifteen years ago Scotter was notorious in the neighbourhood for its fevers, a notoriety mainly earned through the frequent occurrence of epidemics of typhoid fever." . . . "Typhoid fever has almost disappeared; on the other hand pneumonia has greatly increased." . . . "The epidemic (of 1886 and 1890), was entirely a local manifestation. It originated in Scotter, and spread from it as a centre; and it was independent of weather conditions." . . . "The most striking feature in this rush of cases was their enormous fatality and their partial distribution." . . . "Forty houses had ten cases, while all the rest within the parish boundaries, 217 in number, had only the same number." The last outbreak of pneumonia Dr. Eminson attributes entirely to inefficient house and street drainage.

If all England had "modern eyes," all England would be able to see these things in their naked shamefulness. Newspaper writers would see them, and squires, and country parsons, and farmers, and tradesmen, and even the cottagers themselves. But because hardly any of us have modern eyes, but traditional, conventional, ignorant, and foolish eyes, the bulk of us never see these things at all. We do not comprehend them when they are pointed out to us. The squire, and the parson, and still more the smaller ratepayers, look upon the physiologist, who insists upon preaching his gospel of health, as a mere faddist. But it would be better to be a faddist than a blind and obstinate fool. As a matter of fact, however, the physiologist's gospel is as reasonable as the Ten Commandments, and much more demonstrable than nine-tenths of the theology that is preached from the pulpits of modern churches and chapels.

Scotter is a sample of scores of English villages. The East of England, including Lincolnshire, Suffolk, and Essex, seems to be especially antiquated and degraded in its village life. The modern eye has fixed itself upon all this untamed and untaught savagery of life, and the modern spirit has resolved to put an end to it. This is the great task that lies before politicians and other public-spirited men in the immediate future. England has to be vivified with a new life; the vivifying force is science, science that is itself living and generous. The task has got to be performed and completed. If the leaders in science will do it, so much the better; but if not, the younger generation of scientific men will quietly put the older on one side and perform the task themselves.

